# The New Kid on the Block: Validating the Role of Indocyanine Green for Sentinel Lymph Node Biopsy in the Post-neoadjuvant Setting in Patients With Breast Cancer

**DOI:** 10.7759/cureus.81255

**Published:** 2025-03-26

**Authors:** Harpyar Singh, Chintamani Chintamani, Sachin Kolte, Sabyasachi Hazra, R Gokulakrishnan, Hrishikesh MS, Kuozokhotuo Suohu

**Affiliations:** 1 Department of General Surgery, Vardhman Mahavir Medical College and Safdarjung Hospital, New Delhi, IND; 2 Department of Surgical Oncology, Sir Ganga Ram Hospital, New Delhi, IND; 3 Department of Pathology, Vardhman Mahavir Medical College and Safdarjung Hospital, New Delhi, IND

**Keywords:** breast and endocrine surgery, breast conservative surgery (bcs), intraoperative icg, sentinel lymph node biopsy (slnb), thoracic and breast oncology - areas of interest

## Abstract

Background and objective

There is scarce literature on the role of indocyanine green (ICG) in sentinel lymph node biopsy (SLNB) post-neoadjuvant chemotherapy (NACT) in breast cancer patients, specifically in the Indian population. This study aimed to address this gap by evaluating the identification rates and accuracy of SLNB using ICG.

Methods

A prospective observational study was conducted over 18 months at Vardhman Mahavir Medical College, New Delhi, involving 30 patients with locally advanced breast cancer. The primary objectives were identification rates, positive predictive value, and false negatives. All patients underwent SLNB post-NACT and, irrespective of SLNB results, underwent axillary lymph node dissection (ALND). The secondary objectives of the study focused on complications related to ICG dye.

Results

The mean age of the cohort was 44.83 years. The SLNB technique using ICG showed an identification rate of 100% with a sensitivity of 91.67%, a specificity of 100% with a false negative rate of 5.55%, and an accuracy of 96.67%. No cases of allergic reactions to ICG or skin necrosis were observed.

Conclusions

Our findings validate the use of ICG dye for SLNB in neoadjuvant setting for patients with breast cancer, demonstrating high sensitivity and specificity. ICG fluorescence imaging permits real-time visualization of lymphatics for SLNB and, at the same time, minimizes complications and high-tech nuclear medicine infrastructure requirements.

## Introduction

According to GLOBACON 2022 statistics, breast cancer accounts for 11.6% of all cancer diagnoses globally and is responsible for 6.9% of cancer-related deaths [1]. There has been a remarkable shift in breast cancer treatment, from radical mastectomies to breast-conservation surgeries, with sentinel lymph node biopsy (SLNB) now considered the standard of care for early breast cancers [2]. SLNB utilizes various techniques and dyes, such as methylene blue, isosulfan blue, indocyanine green, carbon nanoparticles, and radiotracer colloids, to trace lymphatic pathways. While SLNB can have complications, its associated morbidity is significantly lower than that of level I and II axillary lymph node dissections (ALND), especially regarding issues like upper limb swelling, seroma formation, and shoulder stiffness [3].

A significant drawback of using isosulfan blue and patent blue as tracers in SLNB is the potential for allergic and anaphylactic reactions, which occur in approximately 0.06%-2.7% of cases. Although severe reactions to methylene blue are uncommon, it can lead to persistent skin tattooing and, in rare cases, skin necrosis if injected intradermally [4]. Conversely, the necessary infrastructure for radiocolloids is lacking. Hence, indocyanine green (ICG) dye emerges as a valuable alternative. Its accessibility allows for SLNB even in settings where radiocolloids may not be feasible, with fewer complications and better results in terms of identification rates and positive predictive value. While the SLNB for axillary staging in patients undergoing neoadjuvant therapy is promising, it has yet to be formally recognized as the standard of care.

The existing literature on the effectiveness of SLNB using ICG for axillary staging in Indian population is sparse and role of SLNB in itself is yet to be established in axilla addressal in post-neoadjuvant settings. In light of this, this study aimed to investigate the accuracy of SLNB using ICG for axillary staging in patients with breast cancer after neoadjuvant chemotherapy (NACT), to address the identified gaps in the literature.

## Materials and methods

A prospective observational study was conducted in the Department of Surgery, Vardhman Mahavir Medical College (VMMC), Safdarjung Hospital (SJH), New Delhi, between April 2022 and September 2024. The study received ethical clearance from the Institutional Review Board and the Ethics Committee.

In this study, 30 confirmed breast cancer cases were enrolled after obtaining informed consent. The inclusion criteria included all patients with locally advanced breast cancer undergoing breast surgery with ALND. The exclusion criteria included patients with metastatic breast cancer and allergy to ICG dye. All patients with clinically negative axillary nodes post neoadjuvant chemotherapy underwent SLNB using ICG dye (study technique). Irrespective of the SLNB results, all patients underwent ALND (gold standard). 

**Figure 1:**
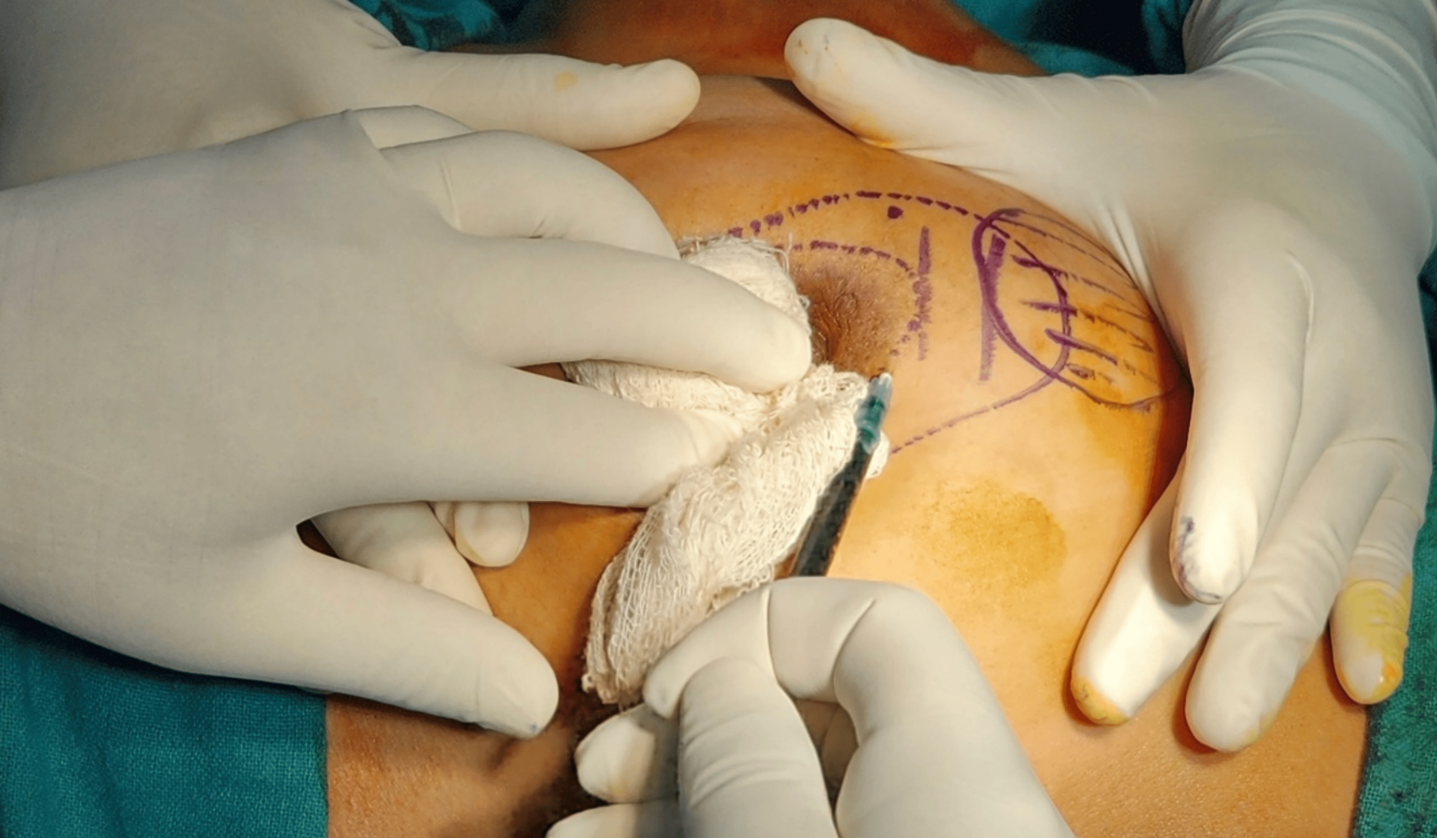
Periareolar instillation of ICG dye ICG: indocyanine green

ICG dye (Aurogreen) was prepared from a vial of ICG dye containing 25 mg dye. Sterile water was injected into the vial, and the solution was prepared after mixing for two to three minutes. The final dye was prepared in a concentration of 2.5 mg/ml and was loaded using a filter system into 2 ml syringes; 3-5 ml of dye was injected into the periareolar area after the patient was intubated (Figure 1).

**Figure 2:**
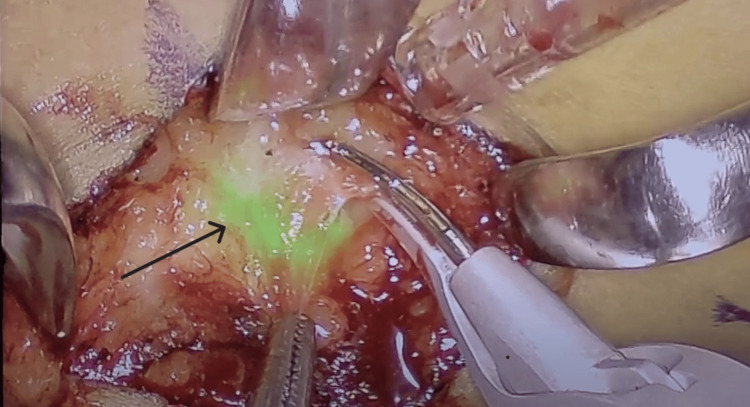
Magnified intraoperative view of the SLN (black arrow) identified by ICG fluorescence ICG: indocyanine green; SLN: sentinel lymph node

This was followed by breast massage for 5-10 minutes. Meanwhile, the imaging system, which consisted of a laparoscopic camera adjusted to ICG dye mode, was set up along with the monitors. The flow of dye through the lymphatics was imaged until it reached the axilla, where it disappeared into a deeper region in the axilla, and then that region was explored for SLN (Figure 2). The differentiation of SLN from second-tier nodes was based on (1) the presence of a lymphatic channel leading to the sentinel node, (2) the sequence of appearance (the sentinel node appears first), and (3) the intensity of uptake (the sentinel node has more activity).

**Figure 3:**
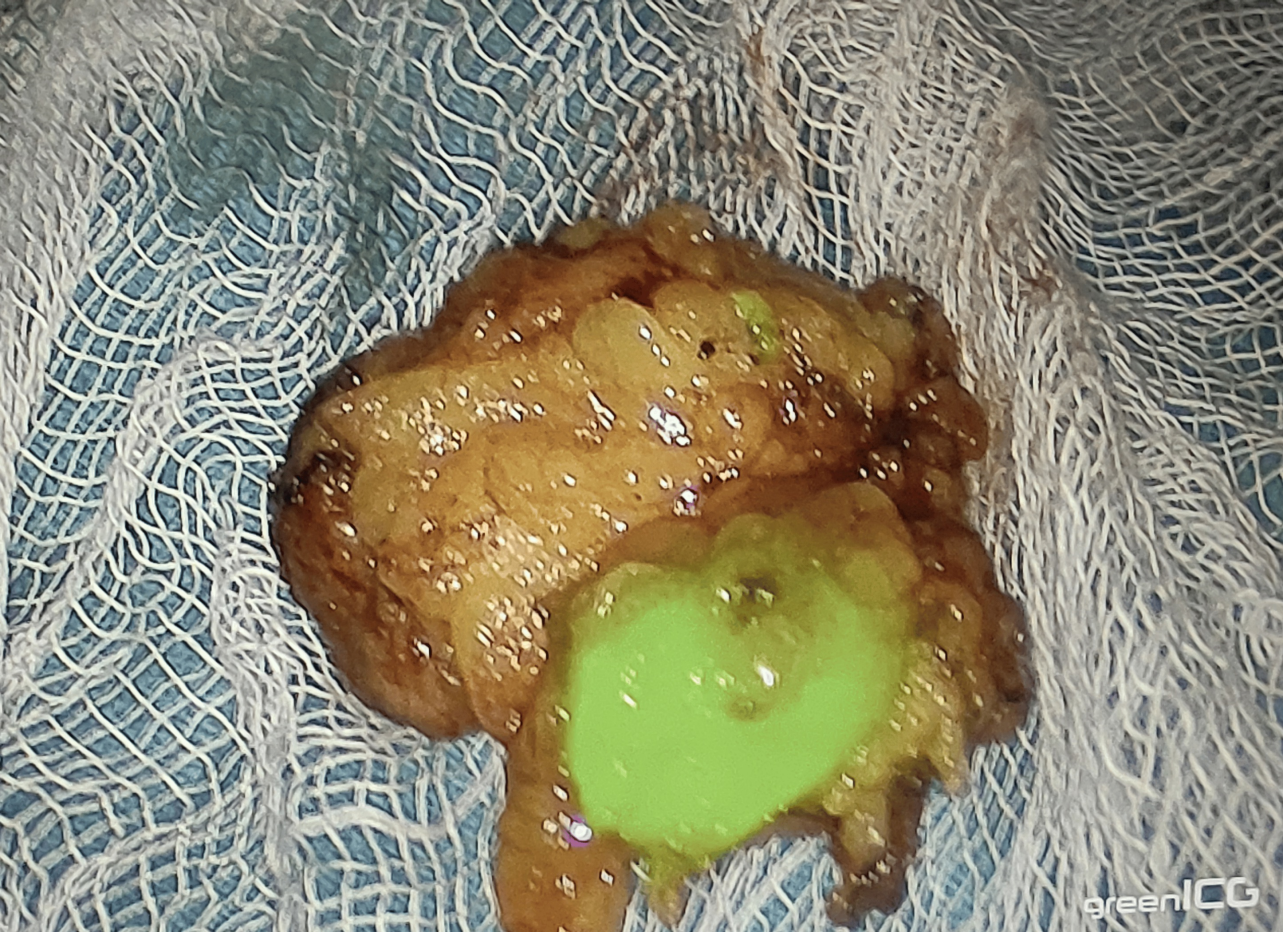
SLN with ICG fluorescence after SLNB, prior to being sent for frozen section analysis ICG: indocyanine green; SLN: sentinel lymph node; SLNB: sentinel lymph node biopsy

The identified SLN (Figure 3) was then sent for the frozen section. Irrespective of the results of the SLN frozen section, all patients underwent ALND, and the specimen was sent for histopathological examination in the department of pathology, VMMC, and SJH. The results of SLN using ICG dye (study technique) and ALND (gold standard) were compared to calculate the sensitivity, accuracy, and false negative rates. Further management of patients was done according to the American Joint Committee on Cancer (AJCC) management guidelines 8th edition. 

Data on demographic characteristics, tumor staging, and histopathological findings were collected. True and false positive cases were identified by comparing SLNB results to ALND results. The injection safety of ICG was calculated in terms of allergic rates to ICG and skin necrosis rates. Statistical analyses were performed using SPSS Statistics verison 29.0 (IBM Corp., Armonk, NY), calculating sensitivity, specificity, negative predictive value, and false negative rate.

## Results

The mean age of participants was 44.83 ± 10.01 years, and the majority (70%) were aged 31-50 years. Most participants had tumors classified as T2 (53.3%), with no metastatic involvement (M0) before NACT. None of the patients had N0 disease or any metastatic deposits (Table 1). Post-NACT, 30% of patients (n=9) had a complete response, and all 30 patients had clinically non-palpable axillary nodes (N0 disease).

**Table 1:** Demographic and clinical characteristics of the study participants BMI: body mass index; NACT: neoadjuvant chemotherapy; NST: no special type

Characteristics		No. of patients (N=30)
Age group, years		
<30		1
31-40		10
41-50		11
51-60		6
>60		2
BMI, Kg/m^2^		
<25		22
>25		8
Pre-NACT TNM staging		
T	1	4
	2	16
	3	10
N	1	22
	2	8
M	0	30
Post-NACT TNM staging		
ycT0N0MO		9
ycT1N0MO		14
ycT2N0MO		7
Histological subtype		
Infiltrating ductal		2
Infiltrating lobular		20
Infiltrating NST		8
Molecular subtype		
Luminal A		6
Luminal B		10
Her2n enriched		7
Triple negative		7

Infiltrating ductal carcinoma was the most common (66.7%) variant, with molecular subtype luminal B (33.0%) as the most common type. Triple-negative cases accounted for 23.3% of cases. The identification rates, which refer to the proportion of patients with a successfully identified SLN out of the total number of patients who underwent SLN biopsy, was 100% in this study.

The outcomes were classified as true positives, true negatives, and false negatives, with the findings of the full ALND serving as the reference standard for comparison. The various terms used are described in Table 2.

**Table 2:** Description of terms used for various outcomes in the study ALND: axillary lymph node dissection; SLN: sentinel lymph node

Variable	Definition	N (N=30)
True positive SLN	A true positive was defined as a positive SLN, and the axilla was also positive	11
False positive cases	A false positive was defined as a positive SLN, and the axilla was negative	0
True negative SLN	A true negative was defined as a negative SLN and a negative axilla after ALND	18
False negative SLN	A false negative SLN was one where the SLN was negative, but the axilla was positive after ALND	1

SLNs were successfully identified in all 30 patients, reflecting a 100% identification rate and demonstrating the reliability of the technique. A total of 108 SLNs were harvested, with an average of 3.6 nodes detected per patient. Among these, 16 SLNs (14.8%) tested positive for malignancy, indicating a notable prevalence of nodal involvement in this patient population.

Of the total 30 patients, the SLN and the axillary nodes were both found to be positive in 11 patients. In contrast, one patient had a negative SLN but a positive axillary node, suggesting that the SLNB did not accurately predict the axillary status. It was a complete response case with pre-NACT staging as T3N1M0 with a lymph node burden of 4/15 in ALND. In 18 patients, both the SLN and axillary lymph nodes were negative, confirming that SLNB reliably predicted axillary status. In all patients, the SNL(s) were located at level I (lateral to the pectoralis minor). The various characteristics of SLNB are tabulated in Table 3.

**Table 3:** Characteristics of SLNB using ICG fluorescence ALND: axillary lymph node dissection; ICG: indocyanine green; SLN: sentinel lymph node; SLNB: sentinel lymph node biopsy

Characteristics	n/N
Identification rates	
SLN identified in patients	30/30
SLNB characteristics	
Total no. of SLN harvested	108
Total positive SLN	16
Mean no. of nodes detected	3.6
No. of patients with positive SLN nodes	11
No. of patients with positive ALND	12
Positive node characteristics	
Micrometastasis	3/11
Macrometastasis	8/11
Isolated tumor cells	0/11

The evaluation of positive sentinel nodes revealed that micrometastases were present in three of the 11 patients with positive SLNs, accounting for 27.3%, while macrometastases were more prevalent, identified in eight of these patients (72.7%). Notably, no isolated tumor cells were detected, indicating the absence of minimal cancerous deposits below the threshold of micrometastases in the assessed nodes. The absence of isolated tumor cells may reflect a distinct pattern of metastatic spread in this cohort, with potential implications for prognosis and therapeutic decision-making. A comparison of SLNB with ALND was done, as shown in Table 4. Statistical methods were applied to calculate several key parameters, as described in Table 5.

**Table 4:** Comparison of SLNB with ALND in the study ALND: axillary lymph node dissection; SLNB: sentinel lymph node biopsy

SLNB	ALND	
	Negative	Positive
Negative	18 (true negative)	1 (false negative)
Positive	0 (false positive)	11 (true positive)

**Table 5:** Statistical variables used in the study analysis ICG: indocyanine green; SLN: sentinel lymph node; SLNB: sentinel lymph node biopsy

Variables	Definition	SLNB using ICG green
Sensitivity	Proportion of true positives relative to the sum of true positives and false negatives	91.67%
Specificity	Proportion of true negatives relative to the sum of true negatives and false negatives	100%
Negative predictive value	Calculated as the proportion of true negatives relative to the sum of true negatives and false negatives	94.73%
False negative rates	Proportion of false negatives to the total number of true negatives	5.55%
Accuracy	Determined by adding the number of true positives and true negatives and dividing them by total no. of patients with a successfully identified SLN	96.67%

There was no false positive case in this study. To sum up, the overall accuracy of the SLNB procedure, computed as the sum of true positives and true negatives divided by the total number of patients with a successfully identified SLN, was found to be 96.67%. No instances of periareolar skin necrosis or allergic reactions to ICG were observed.

## Discussion

SLNB is a widely recognized surgical procedure for patients with early-stage breast cancer [5]. It is associated with lower morbidity and improved quality of life compared to traditional axillary treatments. SLN models illustrate the lymphatic drainage pathway from a primary tumor to the SLN within the regional lymphatic system [6]. If the SLN is cancer-free, performing only SLNB without further ALND is sufficient for patients with negative lymph nodes. This approach minimizes surgical invasiveness and associated complications.

For decades, the dual-dye technique has been the preferred method for SLN detection. This technique has proven highly effective, with false-negative rates ranging from 5% to 10% [7]. Major clinical trials, such as AMAROS [8] and ALMANAC [9], have reported SLN identification success rates of 96% to 97%, establishing dual-dye localization as a reliable standard in SLN detection. In a study involving 847 patients by Sugie et al., the identification rates and sensitivity of SLNB using ICG green were found to be 97.2% and 95.7% [10]. In a recent Indian study by Somashekhar et al. comparing ICG with dual method (methylene blue + radiocolloid) for SLNB involving 100 participants, the identification rates for SLN were found to be 96% with sensitivity and false negative rates of 96.8% and 3.1%, respectively [11]. In our study of 30 patients, the identification rate was 100%, with sensitivity, accuracy, and false negative rates of 91.67%, 96.67%, and 5.55%, respectively.

Previous studies have reported that both obesity and increased age may reduce the success of SLNB [12]. However, in our study, SLN was identified in all 30 cases, irrespective of age and BMI. There were only two cases >60 years of age, and SLN was successfully identified in both cases. There were eight cases with BMI >25 kg/m^2^, and SLN was identified in all eight cases. These findings affirm that SLNB using ICG is a dependable method for axillary evaluation.

The histological evaluation of axillary lymph nodes remains a critical prognostic factor in breast cancer patients, even after receiving NACT [13]. Originally introduced to reduce the size of locally advanced breast cancer (LABC) and enable more effective surgical options, NACT has also been shown to enhance both disease-free survival and overall survival, with outcomes comparable to those of adjuvant chemotherapy [14]. In recent years, the use of NACT has expanded to include certain patients with early-stage disease, allowing for the possibility of breast-conservation surgery [15]. Additionally, one key benefit of NACT is the ability to assess in vivo chemotherapy response, which offers important prognostic insights [16]. Traditionally, ALND has been performed after NACT as part of optimal breast cancer surgery. However, this procedure is associated with significant morbidity [17]. Hence, there is increasing interest in less invasive alternatives, with SLNB after NACT emerging as an appealing option. This is particularly relevant as many patients experience downstaging of the axilla to N0 following NACT.

NACT may theoretically impact the accuracy of SLNB in several ways. First, both the primary tumor and metastatic lymph nodes can undergo reactive changes, such as fibrosis, which can alter lymphatic drainage patterns. Second, chemotherapy may lead to an uneven response in the axillary region, further complicating the assessment. These factors could reduce the accuracy of SLNB following NACT. However, multiple studies have shown that while the identification rates may decrease, the predictive value of SLNB remains largely unaffected after NACT [18-21]. The accuracy and false-negative rates of SLNB after NACT are comparable to those reported in multicenter trials of sentinel node biopsy (SNB) performed without NACT. The false negative rate in this study was 5.55%, consistent with the range observed in pre-NACT SLNB studies. This suggests that concerns regarding skip nodal metastasis may be overstated and that SLNB remains a reliable technique post-NACT.

Safety is a crucial consideration in the adoption of ICG for SLNB. There were no instances of allergic reactions or skin necrosis related to ICG in our study. This favorable safety profile is endorsed by similar findings in the literature, with studies reporting no adverse reactions associated with ICG [10].

Limitations

The major limitation of this study is its small sample size, which restricts the generalizability of the findings to the broader population. Future studies should involve larger cohorts and focus on long-term outcomes to validate these findings further.

## Conclusions

Our findings reinforce the advantages of using ICG in SLNB for breast cancer patients, not only in terms of efficacy and safety but also in improving patient outcomes. Future research should involve larger cohorts to further validate these findings and standardize techniques for optimal lymphatic mapping in clinical practice. The SLNB technique in NACT cases has shown significant potential as a future standard of care, enhancing staging accuracy and minimizing morbidity.
